# A new species of *Lathys* from Turkey (Araneae, Dictynidae)

**DOI:** 10.3897/zookeys.632.10130

**Published:** 2016-11-16

**Authors:** Recep Sulhi Özkütük, Yuri M. Marusik, Mert Elverici, Kadir Boğaç Kunt

**Affiliations:** 1 Department of Biology, Faculty of Science, Anadolu University, TR-26470, Eskişehir, Turkey; 2 Institute for Biological Problems of the North, Portovaya Street 18, Magadan 685000, Russia; 3 Department of Zoology & Entomology, University of the Free State, Bloemfontein 9300, South Africa; 4 Department of Biological Sciences, Faculty of Arts and Sciences, Middle East Technical University, TR-06800 Ankara, Turkey; 5 Department of Biology, Faculty of Science and Arts, University of Erzincan, TR-24100, Erzincan, Turkey

**Keywords:** Aranei, Asia, Central Anatolia, meshweb spiders

## Abstract

A new species, *Lathys ankaraensis* **sp. n.**, is described based on the material collected in the Central Anatolia. The new species belongs to the *humilis*-group. Habitus, as well as copulatory organs of both sexes, are described and illustrated by means of line drawings and digital and SEM photographs. A key for the four *Lathys* species known in Turkey is also provided.

## Introduction

*Lathys* Simon, 1884 with 45 known species, is one of the largest genera of Dictynidae distributed chiefly in the Holarctic (WSC 2016). So far, three species of *Lathys* have been reported from Turkey: *Lathys humilis* (Blackwall, 1855), *Lathys lehtineni* Kovblyuk, Kastrygina & Omelko, 2014, and *Lathys stigmatisata* (Menge, 1869) (Bayram et al. 2016). All species were recently redescribed in details by Marusik et al. (2006, 2009a,b) and Kovblyuk et al. (2014). Recent field studies focused on litter sampling revealed one more species new to science. It was found in several localities from woodland habitats in Central Anatolia.

The goal of this paper is to provide a description of the new species together with notes comparing the two sibling species.

## Material and methods

**Figure 1.**
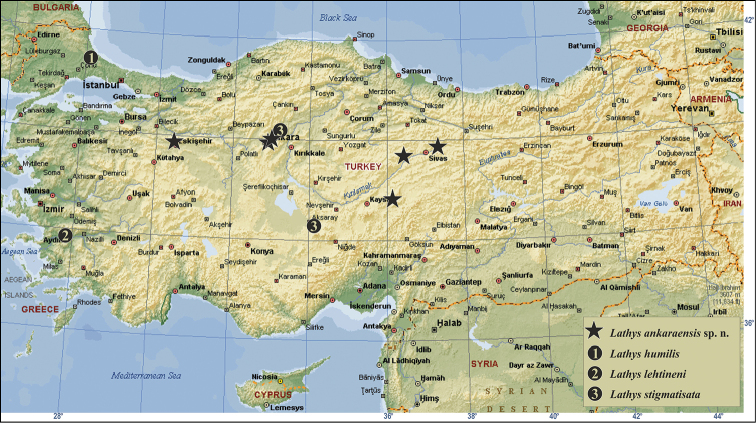
Distribution map of four Turkish *Lathys* spiders.

Examined specimens were collected from the Central Anatolia region of Turkey by using a litter reducer (Fig. 1). The specimens were preserved in 70% ethanol. Digital images of the copulatory organs were taken with a Leica DFC295 digital camera attached to a Leica S8AP0 stereomicroscope and several photographs were taken in different focal planes and combined using auto montage software. SEM microphotographs were made from dried and sputter coated (by gold) organs by use of a Zeiss Ultra Plus SEM device (Anadolu University, Eskişehir). All measurements are in millimeters.

The following abbreviations were used in the text:

Fe
 femur 


Me
 metatarsus 


Pa
 patella 


Ta
 tarsus 


Ti
 tibia 


**Depositories:** AUZM, Anadolu University Zoological Museum (Eskişehir, Turkey); ZMMU, Zoological Museum of Moscow University (Moscow, Russia).

Drawings 8–16 and 17–21 are made by M. Kovblyuk and Z. Kastrygina.

## Taxonomy

### Lathys ankaraensis sp. n.

Taxon classificationAnimaliaAraneaeDictynidae

http://zoobank.org/23A710E1-61A5-4415-B5C6-DC256FAB07B2

Figs 2–5 , 8–11 , 17–19 , 22–28 , 31–35 , 37–40

#### Material examined.

Holotype ♂ (AUZM) Ankara Province, Çankaya Disctrict, Türkkonut, Dodurga Village (40°0'26.01"N; 32°35'23.78"E), 1090 m, 27 May 2012, M. Elverici leg. Paratypes 6♂, 13♀ (AUZM); ♂, 2♀ (ZMMU) same data as holotype; 5♀ (ZMMU) Eskişehir Province, Centrum, Meşelik Area (39°43'25’’N; 30°29'17’’E), 980 m, young pine stand with oak shrubs, 26 September 2010, Y.M. Marusik leg.; 2♀ (ZMMU) Eskişehir Province, Çatacık Forests (39°55'54’’N; 31°08'22’’E), 1190 m, pine stand with few oaks, 27 September 2010, Y.M. Marusik leg.; 2♂ (ZMMU) Ankara Province, Çamlıdere District (40°32'42.54"N; 32°30'0.00"E), 960 m, litter under *Pinus* trees, 28 May 2009, Y.M. Marusik leg.; 2♀ 2 juv. (ZMMU) Ankara Province, Çankaya District, Dodurga Village (39°49'16.20"N; 32°40'5.90"E), 1080 m, shrubby oak stands in steppe, sifting litter, 1 January 2013, Y.M. Marusik leg.; 2♀ (AUZM) Sivas Province, İmranlı District, Yapraklıpınar Village (39°47'52.93"N; 38°5'3.75"E), 1700 m, 14 October 2015, K.B. Kunt leg.; 3♂, 2♀ (AUZM) Sivas Province, Gemerek District, İkizce Village (39°12'52.84"N; 36°10'23.48"E), 1290 m, shrubby oak stands in steppe, 20 November 2015, K.B. Kunt leg.; 2♂, 5♀ (AUZM) Kayseri Province, Pınarbaşı District, Kazancık Village (39°3'41.26"N; 36°33'54.93"E), 1600 m, 29 April 2016, K.B. Kunt leg.

#### Derivatio nominis.

The specific name is a toponym that refers to the type locality, Ankara, capital city of the Republic of Turkey.

#### Diagnosis.

*Lathys ankaraensis* sp. n. belongs to the *humilis* species group represented by two species in the West Palaearctic, *Lathys humilis* (Blackwall, 1855) and *Lathys nielseni* (Schenkel, 1932). It can be distinguished from the congeners by a combination of the following characters: having white guanine spots on dorsum of abdomen (absent in *Lathys nielseni*), longer copulatory ducts (*Cd*) with a series of loops (Figs 18, 24, 39) (single loop in the congeners), partially fused atria (*At*, separated in *Lathys humilis*), wider septum (*Se*) occupying anterior half of fovea (thin and long in *Lathys humilis*), and straight posterior tip of conductor (*Tc*) (slightly bent in *Lathys humilis*, cf. Figs 10 and 14).

**Figures 2–7.**
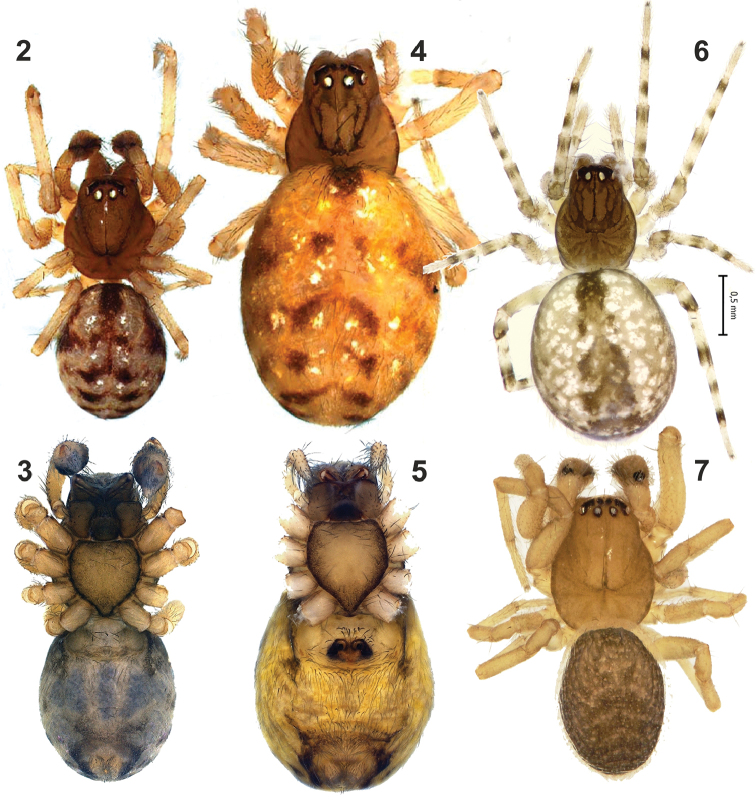
Habitus of *Lathys ankaraensis* sp. n. (**2–5**), *Lathys humilis* (from Turkey **6**) and *Lathys stigmatisata* (from Crimea **7**). **2, 7** male, dorsal **3** male, ventral **4, 6** female, dorsal **5** female, ventral.

**Figures 8–16.**
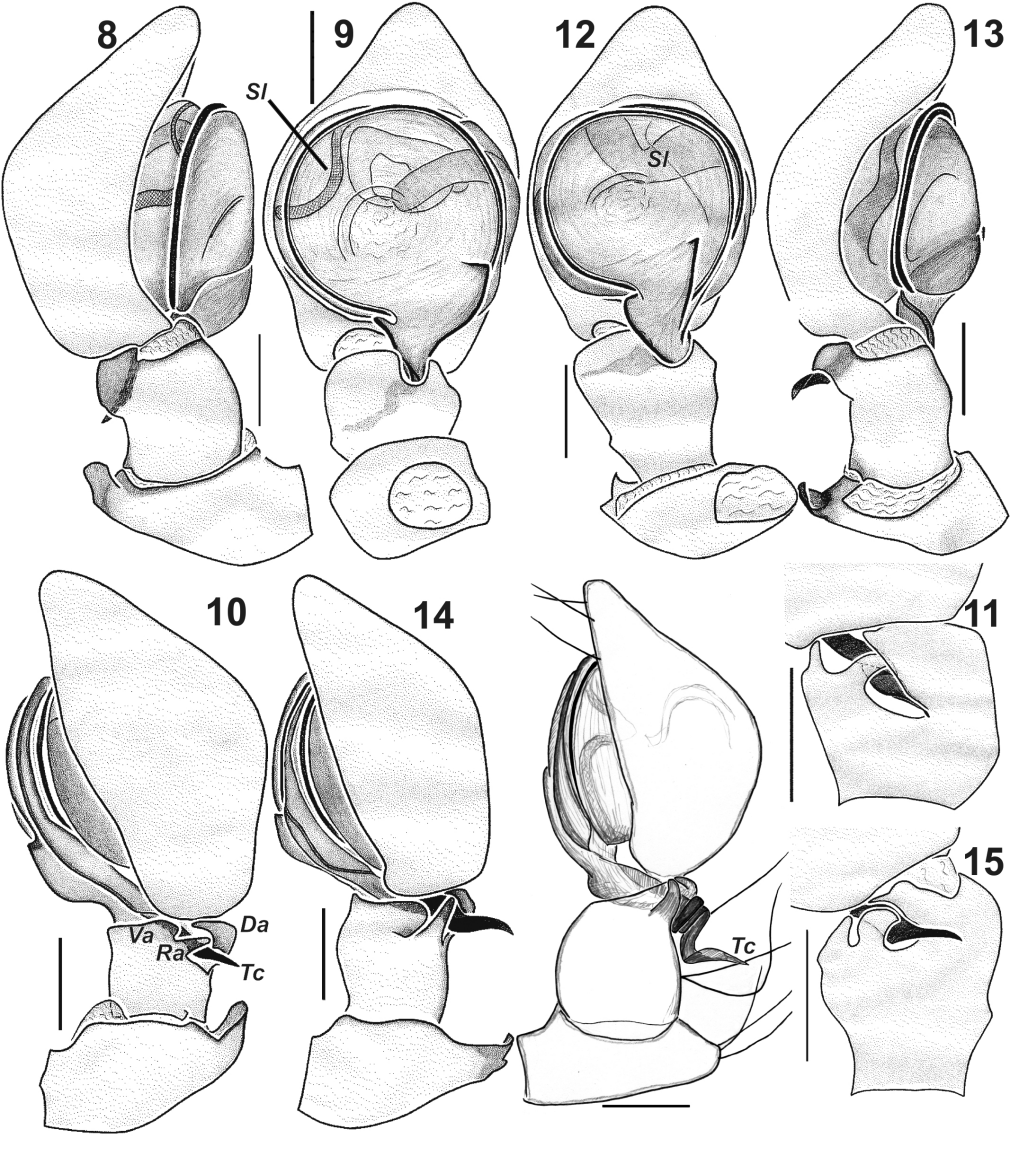
Male palps of *Lathys ankaraensis* sp. n. (**8–11**), *Lathys humilis* (from Turkey **12–15**) and *Lathys stigmatisata* (from Crimea **16**). **8, 13** prolateral **9, 12** ventral **10, 14, 16** retrolateral **11, 15** tibia, tip of conductor and base of cymbium, dorso-retrolateral. Abbreviations: Da dorsal apophysis; Ra retrolateral apophysis; Sl loop of seminal duct; Tc posterior tip of conductor; Va ventral apophysis.

**Figures 17–21.**
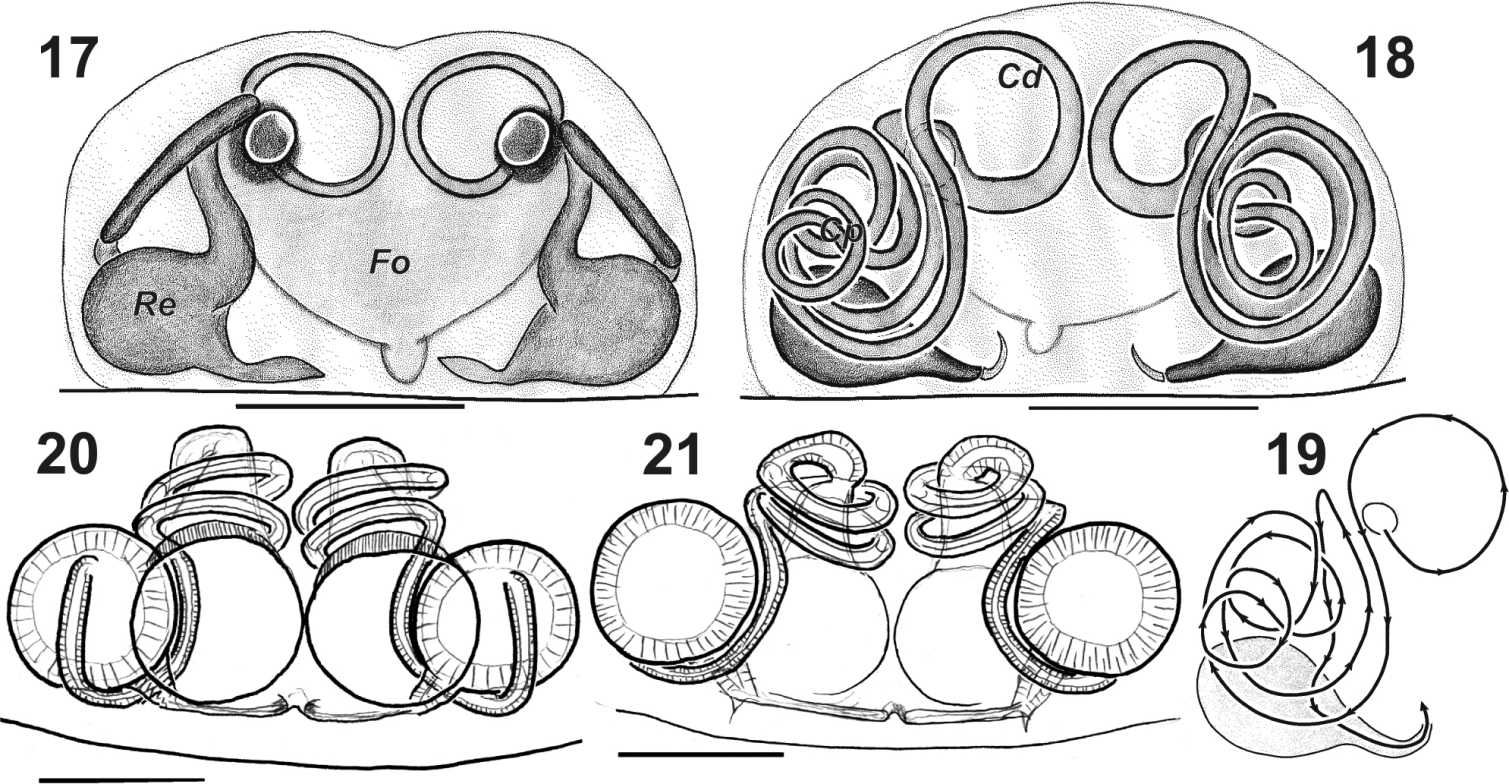
Epigynes of *Lathys ankaraensis* sp. n. (**17–19**) and *Lathys stigmatisata* (from Crimea **20–21**). **17, 20** ventral **18, 21** dorsal **19** schematic drawing of insemination duct. Abbreviations: Cd copulatory ducts; Cp other coils; Fo fovea; Re Reseptacle.

**Figures 22–30.**
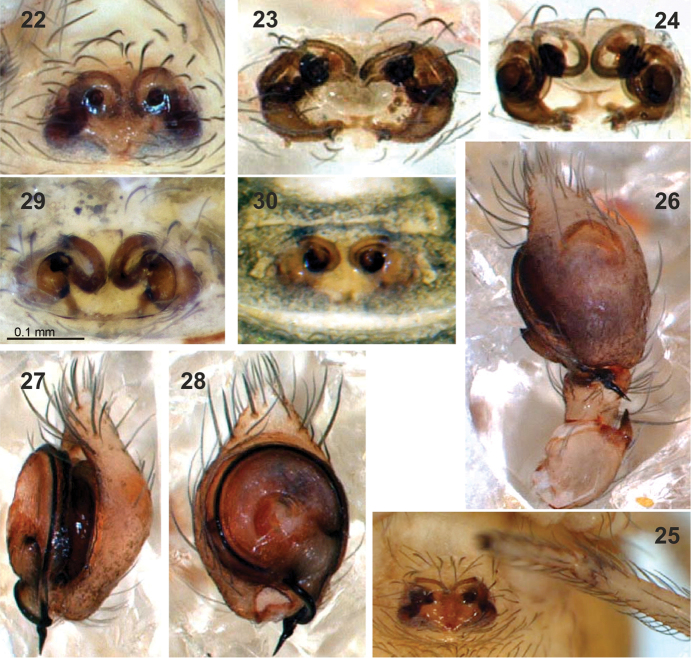
Copulatory organs of *Lathys ankaraensis* sp. n. (**22–28**), *Lathys humilis* (from Turkey **29**) and *Lathys nielseni* (from Finland **30**).

**Figures 31–36.**
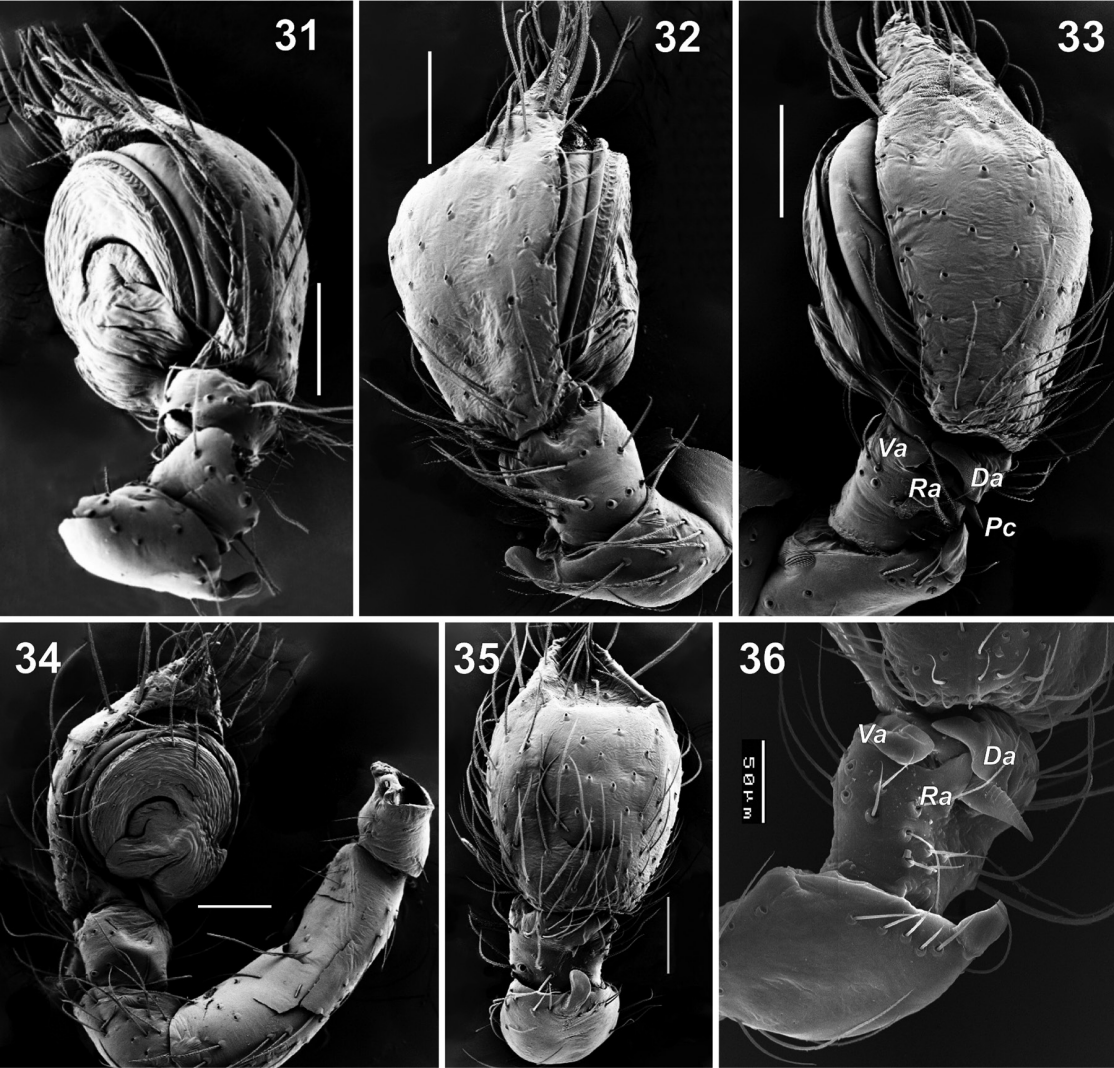
Male palps of *Lathys ankaraensis* sp. n. (**31–35**) and *Lathys stigmatisata* (from Crimea **36**). **31** posterior-retrolateral **32** prolateral **33, 36** dorso-retrolateral **34** ventral **35** dorsal. Abbreviations: Da dorsal apophysis; Pc posterior arm of conductor; Ra retrolateral apophysis; Va ventral apophysis.

**Figures 37–42.**
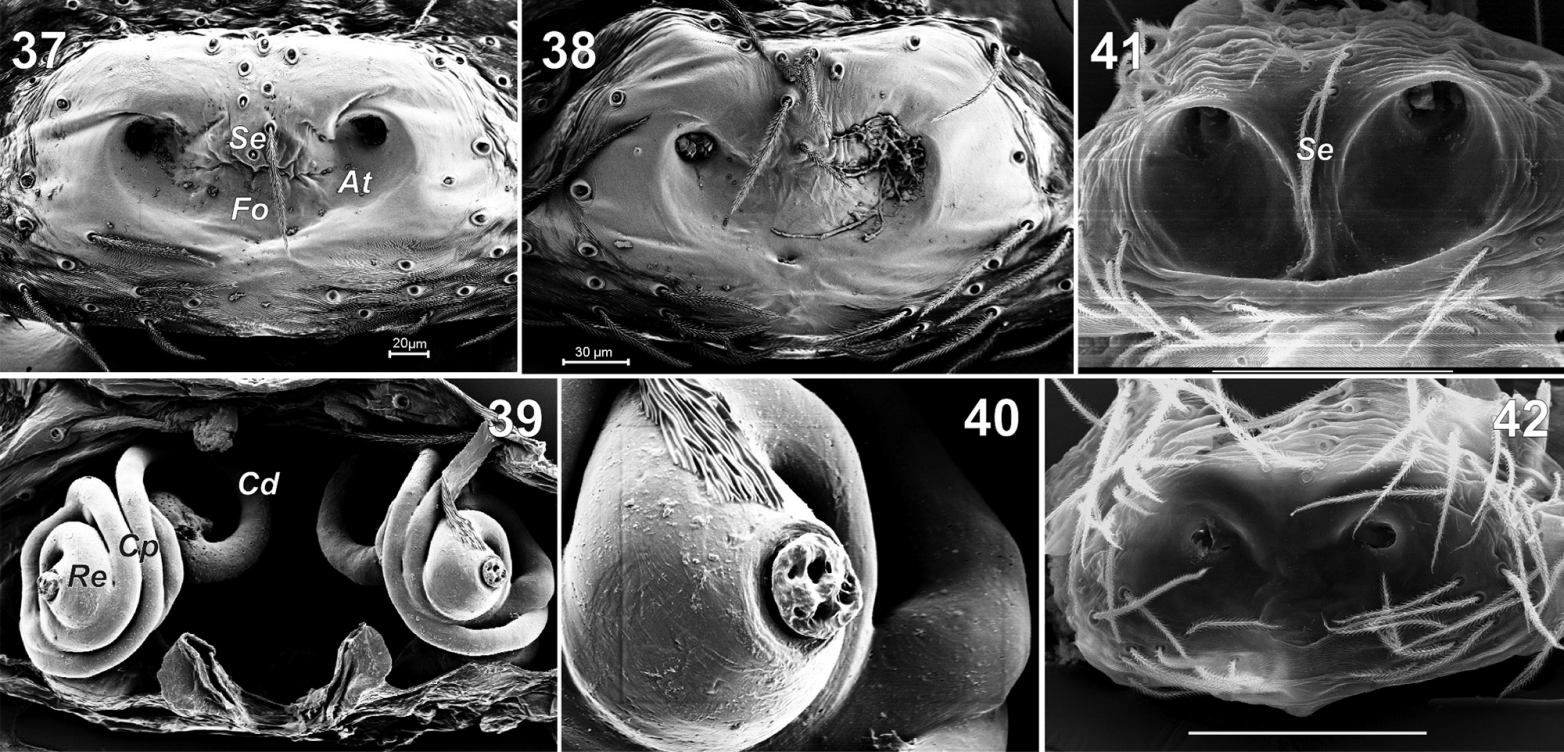
Epigynes of *Lathys ankaraensis* sp. n. (**37–40**), *Lathys humilis* (from Crimea **41**) and *Lathys nielseni* (from Finland **42**). **37–38, 41–42** ventral **39** dorsal **40** receptacle with gland; dorsal. Abbreviations: At atrium; Cd copulatory ducts; Cp posterior coils; Fo fovea; Re Reseptacle; Se septum.

#### Description.

Measurements. **Male.** Holotype ♂: total length 2.00; carapace 1.00 long, 0.72 wide, 0.38 high; chelicerae 0.53 long. Paratypes ♂ (n=9): total length 1.78–2.36; carapace 0.72–1.20 long, 0.66–0.83 wide; 0.35–0.40 high; chelicerae 0.37–0.54 long.

Carapace dark greenishbrown. Cephalic region higher than thoracic region. Fovea distinct, blackish. Darkly colored crack-like pattern with indistinct borders apparent around fovea, at the corners of thoracic region and at the rear side of PME. Anterior eyes arranged in almost straight line. Chelicera color as carapace. Anterior surface of chelicera with irregularly distributed blackish setae of varying sizes, raised on small pits. Anterior margin of the cheliceral groove with four teeth, and posterior margin with three. Teeth on posterior margin smaller than anterior teeth, and almost identical in size to each other. Gnathocoxae yellowish brown, longer than wide, with sparsely distributed tiny, blackish, short setae on the surface. Labium trapezoid, slightly wider than long; darker in color compared to gnathocoxae. Sternum yellowish light brown, dark brown at the edges, with blackish setae on the surface varying in size especially towards the edges. Legs greyish light brown, with blackish setae in all segments, especially intense on ventral surfaces.

Posterior part of segments with dark rings.

Abdomen oval, with a characteristic pattern. Abdominal pattern forming a blackishbrown longitudinal band starting from the middle of the anterior side, barely reaching to the middle of abdomen, followed by five chevrons on the posterior. A variable dark colored pattern apparent onsides; tends to join with the first chevron at the anterior half. Apart from the specified patterns, dorsal side of abdomen grayish light brown, with variably distributed bright white spots. Ventral side of abdomen generally light in color, usually grayish, brown in some specimens.

Palp as in Figs 8–11, 26–28, 31–35; patella with flat dorsal apophysis located on conical dorsal extension; tibia with three apophyses: ventral (*Va*), retrolateral (*Ra*) and dorsal (*Da*); cymbium conical, its height subequal to half of the length; posterior arm of conductor (*Pc*) with almost straight tip locked by three tibial apophyses and cymbium; sperm duct makes a loop (*Sl*) at approximately the 10 o’clock position (Fig. 9).

**Female.** Paratypes ♀ (n=10). Total length 1.90–2.60; carapace 0.54–0.80 long, 0.52–0.56 wide, 0.29–0.40 high; chelicerae 0.25–0.36 long.

Females slightly lighter than males. Crack-like blackish pattern on carapace much more distinct in females. Dorsal pattern on abdomen distinct but usually duller in color compared to males. Calamistrum with eight setae, slightly longer than half of metatarsus. Spines lower in number compared to males. For leg measurements see Table 1.

**Table 1.** Leg measurements of *Lathys ankaraensis* sp. n.

Legs	Fe	Pa	Ti	Me	Ta	Total
♂ / ♀						
I	0.83 / 0.66	0.33 / 0.27	0.75 / 0.52	0.63 / 0.43	0.33 / 0.29	2.87 / 2.15
II	0.70 / 0.59	0.30 / 0.24	0.62 / 0.44	0.54 / 0.36	0.32 / 0.26	2.48 / 1.88
III	0.61 / 0.50	0.26 / 0.22	0.40 / 0.32	0.48 / 0.32	0.30 / 0.22	2.05 / 1.55
IV	0.67 / 0.63	0.27 / 0.25	0.58 / 0.47	0.51 / 0.46	0.30 / 0.26	2.33 / 2.04

Epigyne as in Figs 17–19, 22–25, 37–40; fovea (*Fo*) wide, twice as wide than long, with two partly fused atria (*At*), septum (*Se*) located in anterior half of fovea, wide, covered with few setae. Endogyne with small receptacles (*Re*) and long copulatory ducts forming several coils in two plains, anterior part with one coil (*Cl*) almost parallel to the epigynal plate, and other coils (*Cp*) make several loops around receptacles.

#### Natural history.

It seems that adult specimens of the new species can be found throughout the whole year. *Lathys ankaraensis* sp. n. was found exclusively in the litter under pine trees or oak bushes (Fig. 43).

#### Comments.

The first record of the genus *Lathys* from Turkey has been provided with *Lathys humilis* from the Marmara region (Tekirdağ province; European part of Turkey; van Helsdingen 2013). Subsequent records were presented more recently as *Lathys lehtineni* and *Lathys stigmatisata* respectively from the Aegean (Aydın Province, Danışman et al. 2014) and Central Anatolia (Koçyiğit et al. 2016) regions.

With description of *Lathys ankaraensis*, the number of *Lathys* species known from Turkey has increased to four and number of dictynid species to twenty (Bayram et al. 2016). These numbers are expected to increase in near future as there are species known from the close vicinity such as *Lathys cambridgei* (Simon, 1874), *Lathys spasskyi* Andreeva & Tyshchenko, 1969 or *Lathys nielseni* (Schenkel, 1932), which have a wide distribution in the West Palaearctic. It is worth mentioning that records of *Lathys lehtineni* (Danışman et al. 2014) from Aydın and *Lathys stigmatisata* (Koçyiğit et al. 2016) from Niğde, Aksaray provinces may refer to another species. According to the original description, *Lathys lehtineni* lacks any pattern, but Fig. 3A in Danışman et al. (2014) displays a distinct pattern. A key feature of the male of *Lathys stigmatisata* is the conical outgrowth of the palpal patella (Marusik et al. 2009b), and such outgrowth is missing on Fig. 2 in Koçyiğit et al. (2016).

Below a key to the species reported from Turkey is provided.

### Key to *Lathys* species reported from Turkey

**Males**

**Table:** 

1	Abdomen with white guanine spots, patella with dorsal apophysis, the tip of conductor straight	**2**
–	Abdomen without guanine spots, patella without apophysis, tip of conductor coiled	**3**
2	Tegulum with a prolateral-anterior thin loop of seminal duct (Fig. 9)	***Lathys ankaraensis* sp. n.**
–	Tegulum with an anterior broad loop of seminal duct (Fig. 12)	***Lathys humilis***
3	Palpal patella with dorsal conical outgrowth (Fig. 16), tip of conductor coiled with terminal loop wider than conductor	***Lathys stigmatisata***
–	Palpal patella without dorsal conical outgrowth, the terminal loop of conductor not wider than other loops	***Lathys lehtineni***

**Females**

**Table:** 

1	Abdomen with white guanine spots, epigyne with one atrium, copulatory openings widely spaced	**2**
–	Abdomen without white guanine spots, epigyne without atrium, but with two separate openings	**3**
2	Atrium with a septum (Fig. 41), insemination ducts short, not encircling receptacle	***Lathys humilis***
–	Atrium without septum (Figs 37–38), insemination ducts long, encircling receptacles (Fig. 18, 24, 39)	***Lathys ankaraensis* sp. n.**
3	Copulatory openings small, spaced by approx. 1/2 diameters	***Lathys lehtineni***
–	Copulatory openings large, separated by thin septum (Fig. 20)	***Lathys stigmatisata***

## Supplementary Material

XML Treatment for Lathys ankaraensis
